# Pleomorphic Variants of *Borreliella* (syn. *Borrelia*) *burgdorferi* Express Evolutionary Distinct Transcriptomes

**DOI:** 10.3390/ijms24065594

**Published:** 2023-03-15

**Authors:** Nina Čorak, Sirli Anniko, Christina Daschkin-Steinborn, Viktoria Krey, Sara Koska, Momir Futo, Tin Široki, Innokenty Woichansky, Luka Opašić, Domagoj Kifer, Anja Tušar, Horst-Günter Maxeiner, Mirjana Domazet-Lošo, Carsten Nicolaus, Tomislav Domazet-Lošo

**Affiliations:** 1 Laboratory of Evolutionary Genetics, Division of Molecular Biology, Ruđer Bošković Institute, Bijenička Cesta 54, HR-10000 Zagreb, Croatia; 2 BCA-Research, BCA-Clinic Betriebs GmbH & Co. KG, D-86159 Augsburg, Germany; 3 Institute of Cancer Therapeutics, Faculty of Life Sciences, University of Bradford, Bradford BD7 1DP, UK; 4 Physics of Synthetic Biological Systems-E14, Physics Department and ZNN, Technische Universität München, D-85748 Garching, Germany; 5 Faculty of Electrical Engineering and Computing, University of Zagreb, Unska 3, HR-10000 Zagreb, Croatia; 6 School of Medicine, Catholic University of Croatia, Ilica 242, HR-10000 Zagreb, Croatia; 7 Faculty of Pharmacy and Biochemistry, University of Zagreb, A. Kovačića 1, HR-10000 Zagreb, Croatia; 8 Comlamed, Friedrich-Bergius Ring 15, D-97076 Würzburg, Germany

**Keywords:** transcriptomes, *Borreliella*, *Borrelia*, morphotypes, RNAseq, evolution, pleomorphic variants, phylostratigraphy, orphan genes

## Abstract

*Borreliella* (syn. *Borrelia*) *burgdorferi* is a spirochete bacterium that causes tick-borne Lyme disease. Along its lifecycle *B. burgdorferi* develops several pleomorphic forms with unclear biological and medical relevance. Surprisingly, these morphotypes have never been compared at the global transcriptome level. To fill this void, we grew *B. burgdorferi* spirochete, round body, bleb, and biofilm-dominated cultures and recovered their transcriptomes by RNAseq profiling. We found that round bodies share similar expression profiles with spirochetes, despite their morphological differences. This sharply contrasts to blebs and biofilms that showed unique transcriptomes, profoundly distinct from spirochetes and round bodies. To better characterize differentially expressed genes in non-spirochete morphotypes, we performed functional, positional, and evolutionary enrichment analyses. Our results suggest that spirochete to round body transition relies on the delicate regulation of a relatively small number of highly conserved genes, which are located on the main chromosome and involved in translation. In contrast, spirochete to bleb or biofilm transition includes substantial reshaping of transcription profiles towards plasmids-residing and evolutionary young genes, which originated in the ancestor of *Borreliaceae*. Despite their abundance the function of these *Borreliaceae*-specific genes is largely unknown. However, many known Lyme disease virulence genes implicated in immune evasion and tissue adhesion originated in this evolutionary period. Taken together, these regularities point to the possibility that bleb and biofilm morphotypes might be important in the dissemination and persistence of *B. burgdorferi* inside the mammalian host. On the other hand, they prioritize the large pool of unstudied *Borreliaceae*-specific genes for functional characterization because this subset likely contains undiscovered Lyme disease pathogenesis genes.

## 1. Introduction

Morphological plasticity is the ability of individual bacterial cells to dynamically change their shape in response to environmental conditions [1]. This feature can be found among various bacterial taxa, including pathogens, where colonization of distinct tissues, transmission between hosts, and transit through environmental reservoirs are often accompanied by morphological transformations of bacterial cells [2].

For instance, morphogenic changes in *Legionella pneumophila* are induced by transition from extracellular to intracellular environment as well as by changes in nutrient availability once the bacterium enters the host cell [3,4,5]. Similarly, *Caulobacter crescentus* differentiates into two morphologically distinctive cell shapes as a response to nutrient availability [6]. Another example of morphological plasticity is an uropathogenic strain of *Escherichia coli* which assembles into long filaments in order to evade phagocytosis during infection [2]. These types of morphological changes are often constitutive parts of bacterial life cycles that are underpinned by differential gene expression [2,3,4,6].

In some cases, several morphological forms can be simultaneously present in a bacterial culture at a given time. This population-level phenomenon is often referred to as pleomorphism [1], and is well described in *Escherichia coli*, *Pseudomonas aeruginosa*, *Mycobacterium tuberculosis*, *Salmonella enterica* and *Staphylococcus aureus* [7,8,9]. Such morphological heterogeneity, which includes the presence of stress-tolerant persisters and viable but nonculturable (VBN) cells, often enables selective benefits to bacterial populations under stressful conditions [10]. However, these stress-tolerant cell types are often characterized by low metabolic activity and low replication rates [11]. For this reason, commonly used antibiotics targeting metabolic production have a low impact on the fitness of bacterial populations showing pleomorphism [11]. Additionally, another side-effect of slow metabolism and low replication rates is that stress-tolerant cell types are usually hard to culture in a laboratory [10].

An important example of bacterial pathogen that shows pleomorphism and the existence of stress-tolerant cell types is *Borreliella* (syn. *Borrelia*) *burgdorferi* [12,13,14]. This bacterium is the causative agent of Lyme disease, which is the most prevalent vector-borne disease in the Northern Hemisphere [15]. The versatile life cycle of this pathogen includes the mammalian reservoir as well as the tick vector [16], whose rapid spread in natural ecosystems causes the increase in Lyme disease occurrence [17,18]. Although antibiotic treatments are generally effective against *B. burgdorferi*, about 10–20% of patients develop so-called Post Treatment Lyme Disease Syndrome [12,19]. It has been proposed that this phenomenon may be linked to the presence of persisters in the bacterial population [20]. Nevertheless, a definitive proof is still missing [19]. Some authors further hypothesized that these persister cells correspond to alternative morphotypes of *B. burgdorferi* [12,14]. This idea is supported by the finding that several pleomorphic variants including spirochetes, round bodies, bleb forms, and biofilms can be simultaneously present in *B. burgdorferi* cultures grown in the BSK-II medium—the most common medium used in *B. burgdorferi* cultivation [13]. However, from the phylogenetic perspective, this pleomorphism is not unique to *B. burgdorferi* because other spirochaetes show similar morphotypes [21,22,23].

The spirochete morphotype is the pleomorphic variant of *B. burgdorferi* that shows planar wave morphology. It represents the prevailing morphotype in BSK-II medium raised cultures [13,24,25], which cell envelope consists of the protoplasmic cylinder covered by two lipid membranes and the periplasmic space between them [26,27]. The flagella are located in the periplasmic space between the inner and outer membrane where they contribute to maintaining the planar wave morphology of spirochetes [13,28]. The planar wave shape of this morphotype is an important feature that facilitates bacterial dissemination and host tissue penetration [2,29]. In comparison to alternative pleomorphic forms, the spirochete morphotype is relatively easy to cultivate under laboratory conditions, and thus it is the most commonly studied *B. burgdorferi* cell type [20].

Spherical *B. burgdorferi* cells with intact and flexible cell envelope enclosing numerous flagella are often termed as “round bodies” [13,25]. This N-acetylglucosamine polysaccharide rich morphotype makes up a small subpopulation in BSK-II medium raised cultures [13]. The exposure of spirochetes to distilled water is the commonly used method for the induction of round body morphotype under laboratory conditions [13,25,30]. Withdrawal of rabbit serum from BSK-II medium [31,32], addition of human serum to BSK-II medium [13], antibiotic treatment [12,33] and cultivation in human cerebrospinal fluid [34], monocyte [13], astrocyte [30], or tonsillar tissue laboratory cultures [35] are other conditions that significantly enrich *B. burgdorferi* cultures with the round body morphotype. Moreover, spherical structures with round body morphology were also found in vivo, both in the cerebral cortex of patients with chronic Lyme neuroborreliosis [30] and in the skin tissues of patients with erythema migrans [36].

The least studied pleomorphic form, the so called “bleb” morphotype, is characterized by the formation of outer membrane vesicles (OMVs) on the surface of *B. burgdorferi* cells [25,37]. In *B. burgdorferi,* similar to other bacterial species, these vesicles are carrying diverse cargo such as proteins, DNA, and RNA molecules [38,39]. The bleb morphotype makes up to 4% of *B. burgdorferi* cells raised in the BSK-II culture at 37 °C [13], but a significantly larger percentage of bleb cells can be induced in vitro by other environmental triggers such as antibiotics, components of the complement system and culture aging [24,33]. Additionally, the bleb morphotype was also observed in vivo, in cell cultures isolated from erythema migrans lesions on the skin of Lyme disease patients [33]. Although the role of bleb morphotype in the initiation of autoimmune reactions is proposed [40], its biological significance in these processes still remains largely unknown [13].

The *B. burgdorferi* biofilms are multicellular assemblies composed of spirochete, round body, and bleb cells embedded in a self-produced extracellular polysaccharide matrix [13]. The existence of *B. burgdorferi* biofilms in vitro was confirmed by detection of typical biofilm markers: alginate, calcium and extracellular DNA [41]. Additionally, atomic force microscopy showed that structural rearrangements are taking place at different stages of biofilm development and that channel-like structures are present in *B. burgdorferi* biofilms [41]. In comparison, these features are shown to be a signature of a true developmental process in *Bacillus subtilis*, a well-established biofilm model [42,43]. *B. burgdorferi* biofilms could be raised under various laboratory conditions [13,41], and are also observed in vivo in the brain, heart, liver, kidney [44] and the skin tissues of infected patients [45].

Despite the accumulated evidence that *B. burgdorferi* pleomorphic forms are a biological reality, their role in Lyme disease pathogenesis is still unclear [14,46]. Previous studies examined some biochemical [13,31,47,48] and structural features [13,25] of *B. burgdorferi* morphotypes. However, global expression analyses of *B. burgdorferi* morphotypes are essentially non-existent, apart from the protein profiling of spirochetes and round bodies by 2D gel electrophoresis [13,31]. To address this void in understanding transcriptional changes associated with *B. burgdorferi* pleomorphic forms, we separately grew spirochete, round body, bleb, and biofilm-dominated cultures *in vitro*, harvested their RNA, and recovered their transcriptomes by RNAseq profiling. In addition, to discern evolutionary imprints of differentially expressed genes, we also traced the evolutionary age of *B. burgdorferi* genes by the phylostratigraphic approach [42,49,50,51,52]. Our results revealed distinct transcription profiles and evolutionary imprints that underlie *B. burgdorferi* morphotypes.

## 2. Results

### 2.1. B. Burgdorferi Morphotypes Show Distinct Transcription Profiles

**Figure 1:**
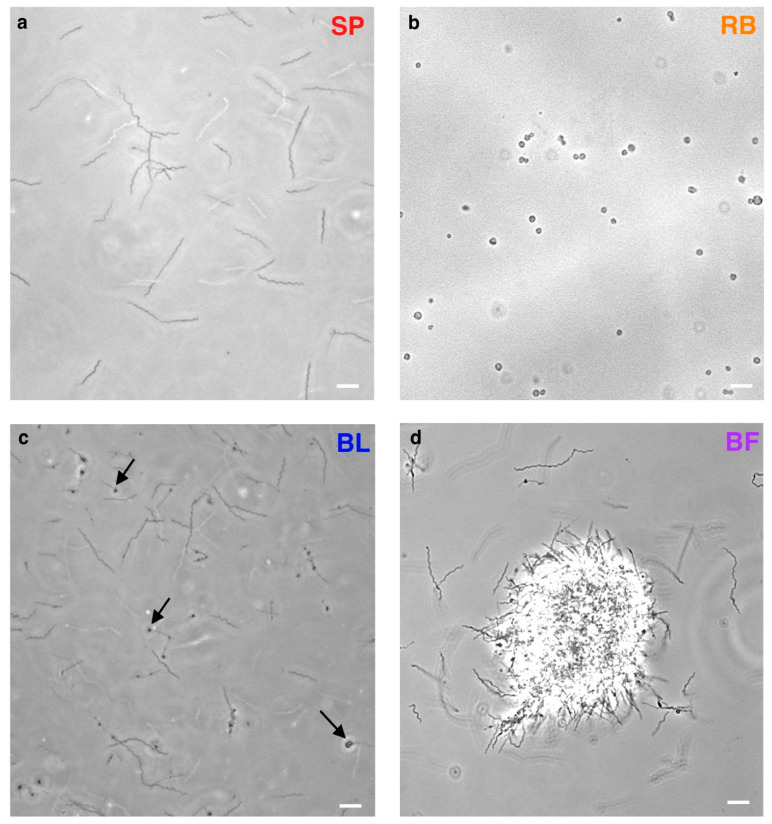
Representative images of *B. burgdorferi* B31 morphotypes. Phase contrast images of *B. burgdorferi* live cell cultures: (**a**) spirochetes (SP), (**b**) round bodies (RB), (**c**) blebs on spirochetes (BL) marked by black arrows and (**d**) biofilm (BF). White bars—10 μm (400× magnification).

**Figure 2:**
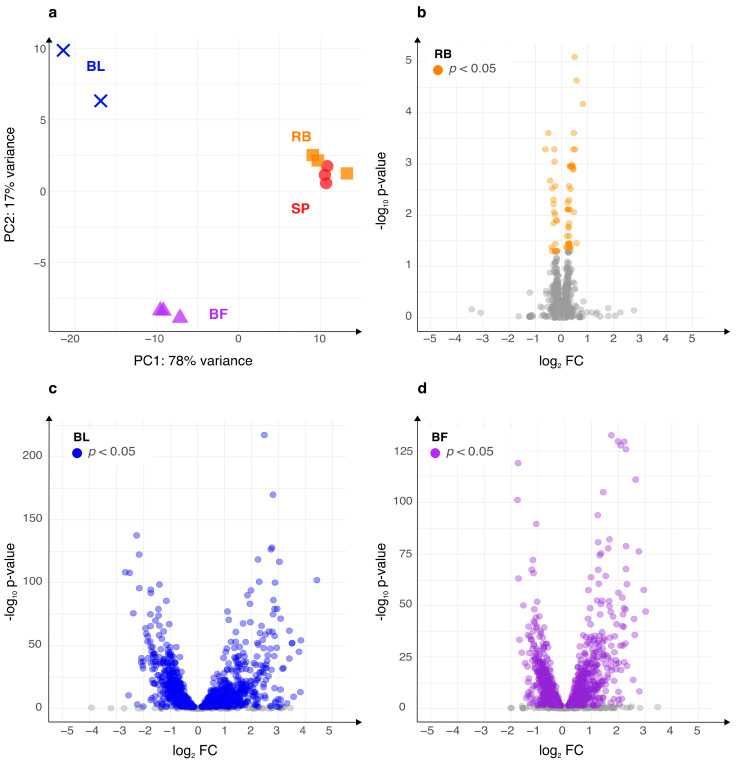
*B. burgdorferi* morphotypes are showing differential gene expression. Spirochete (SP) and round body (RB) dominated cultures share similar expression profiles, while the bleb (BL) and biofilm (BF) dominated cultures show very distinct transcriptomes. (**a**) Principal component analysis (PCA) of *B. burgdorferi* B31 morphotype transcriptome data. The replicates have the same color and symbol. Volcano plots show differentially expressed genes in pairwise comparisons: (**b**) the round body (RB) morphotype in comparison to spirochetes, (**c**) the bleb morphotype (BL) in comparison to spirochetes, and (**d**) biofilms (BF) in comparison to spirochetes. Genes that are significantly differentially expressed (*p*-value < 0.05) are shown in orange (RB), blue (BL), and purple (BF). Genes that are not significantly differentially expressed are shown in gray. PCA and the significance of differential expressions adjusted for multiple comparisons were calculated using the DESeq2 package.

To obtain the transcriptome expression levels of pleiomorphic forms, we induced and sampled independent *B. burgdorferi* B31 cultures where spirochete (SP), round body (RB), bleb (BL), and biofilm (BF) morphotypes strongly predominate (Figure 1, see Section 4). When we considered all four morphotype-dominated cultures together, we found the evidence of transcription for 1370 (89%) predicted *B. burgdorferi* genes. Of these genes, 1306 (92%) were protein coding (see Section 4, Table S1). These numbers are comparable to previous transcriptomic studies in *B. burgdorferi* [38,53,54]. A principal component analysis (PCA) revealed a fairly resolved pattern where biofilm and bleb-dominated cultures have clearly distinct transcriptomes between each other, and compared to spirochete and round body morphotypes that cluster together (Figure 2a).

To identify differentially expressed genes between morphotypes, we compared round body, bleb, and biofilm-dominated cultures against spirochetes (Table S2). We chose these types of pair-wise comparisons since all alternative morphotypes here studied are derived from spirochete cultures after we had implemented specific changes in growth conditions (see Section 4). The magnitude of biologically-relevant expression change is dependent on a gene in focus, and varies largely across the genome. To cover these transcription dynamics, it is generally useful to look for biological patterns at different stringency levels [42]. For this reason, we determined two cut-offs. At the first level we considered all statistically significant differentially expressed genes regardless of their fold-change (permissive criteria), while at the second level we considered only statistically significant differentially expressed genes which had fold-change greater then two (stringent criteria) (Table 1). The comparison of the fold-change and *p*-values in volcano plots reveals that round bodies have a small number of differentially transcribed genes (4.3%, Figure 2b, Table 1). Moreover, the magnitude of fold-change for these differentially transcribed genes is below two-fold (Figure 2b, Table 1). These values show that the transcriptional profile of *B. burgdorferi* round bodies greatly resembles the profile observed in spirochetes, in line with previous work that detected only 77 differentially expressed proteins by 2D gel electrophoresis during spirochete to round body transition [13].

In contrast to round bodies, we detected a high number of differentially transcribed genes in bleb (68%) and biofilm (60%) morphotypes (Figure 2c,d, Table 1). When we applied a more stringent criteria by considering only differentially expressed genes with the magnitude of fold-change above two-fold, we still detected a substantial number of differentially expressed genes (27% blebs, 14% biofilms, Figure 2c,d, Table 1). Our PCA analysis of all genes (Figure 2a) indicated that the bleb and biofilm morphotype express different transcriptomes, hence we tested how many differentially expressed genes are shared between the two morphotypes (Table S3). We found that roughly 70% of differentially expressed genes in biofilms were also differentially expressed in the same direction in the bleb morphotype (Table S3). Combined, this indicates that although bleb and biofilm-dominated cultures have generally distinct transcriptomes, they share a significant proportion of differentially expressed genes.

### 2.2. Morphotype-Specific Functional Enrichments

**Figure 3:**
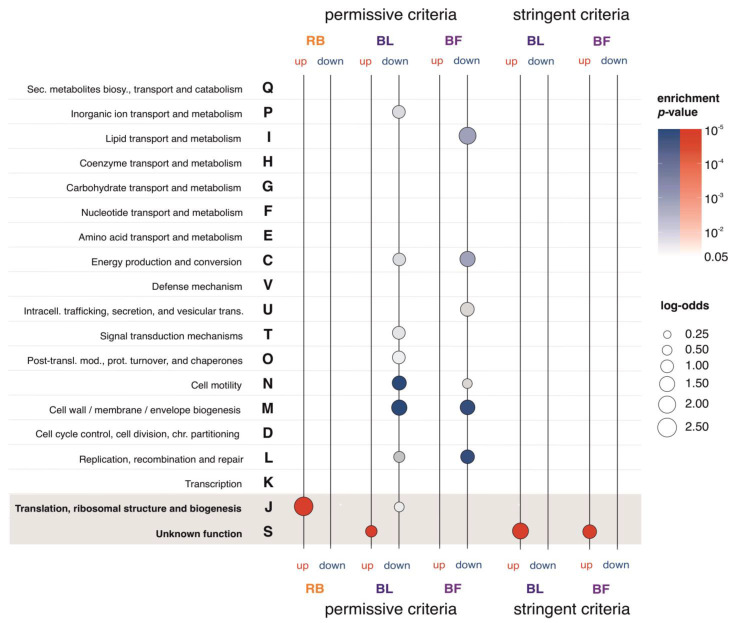
The functional enrichment analysis of differentially expressed genes in *B. burgdorferi* morphotype-dominated cultures. We showed the enrichment profiles of COG functional annotations for upregulated (up, red color) and downregulated (down, blue color) genes that we detected in the round body (RB), bleb (BL), and biofilm (BF) dominated cultures. We tested the significance of enrichment by two-tailed hypergeometric test corrected for multiple comparisons at 0.05 level (Table S5). Differentially expressed genes were determined in reference to spirochetes using DeSeq2 pairwise comparisons. Under permissive criteria, we considered a gene to be differentially expressed if the shift in its expression was statistically significant (*p* < 0.05). Under stringent criteria, we additionally required that the magnitude of change was at least twofold. Under the stringent criteria, there were no differentially expressed genes in round bodies, thus the enrichment analysis was not performed. The magnitude of functional enrichments is depicted by log-odds (circles of different sizes) and their significance is shown in color shades (*p*-values). The gray shaded area marks functions with significant enrichments in upregulated genes across morphotypes.

To detect possible functional trends among differentially expressed genes, we performed a functional enrichment analysis. To achieve this, we first annotated all *B. burgdorferi* genes with COG terms using eggNOG mapper (see Methods). This procedure returned 635 (41%) *B. burgdorferi* genes with at least one functional annotation other than unknown function; i.e., COG term S (Table S13). The round body functional enrichment analysis revealed that only the COG term J (translational, ribosomal structure, and biogenesis) is enriched among round body upregulated genes (Figure 3, Table S5). Interestingly, we found that 22 out of 44 (50%) round body upregulated genes were annotated with COG term J (Table S2). All these 22 genes are coding for structural components of the bacterial ribosome, which makes 40% of all constitutive riboproteins in *B. burgdorferi* (Table S6). Under the assumption that these transcriptional changes are also reflected in protein concentrations [55], the high percentage of riboproteins that are differentially expressed in spirochete to round body transition might relate to ribosome heterogeneity; a phenomenon where changing environmental factors induce the shift in the protein composition of ribosomes [56,57].

The functional enrichment analysis revealed that only COG term S (unknown function) is significantly enriched among bleb upregulated genes (Figure 3, Table S2). Following permissive criteria (Table 1), we found that 356 (67%) genes upregulated in blebs are of unknown function (COG term S). When we applied a more stringent criteria (Table 1 and Table S2, Figure 3), which requires the magnitude of fold-change to be above twofold, we found that 229 (84%) genes upregulated in blebs have unknown function. These surprisingly high numbers of genes with unknown function among bleb-upregulated genes demonstrate that our understanding of the molecular foundations of the bleb morphotype is at present very poor.

In contrast to upregulated genes, the enrichment profiles of downregulated genes in blebs are more diverse (Figure 3, Table S5). For instance, by using the permissive criteria (Table 1) we found that 74% of genes (39 out of 53 genes in the whole genome) which are annotated with the COG term M (cell wall/membrane/envelope biogenesis) are downregulated in blebs (Figure 3, Table S2). This is indicative because blebs are characterized by the formation of large bulges on the outer *B. burgdorferi* membrane [25]. Similarly, 68% of genes (36 out of 53 genes in the whole genome) annotated with the COG term N (cell motility) are downregulated in blebs (Figure 3, Table S2). Again, this is suggestive because it points to the possibility that molecular mechanisms involved in the movement of blebs are different from those governing spirochete movement. However, when we applied a more stringent criteria there were no enriched COG terms in bleb downregulated genes (Figure 3, Table S5). This points to the fact that the magnitude of downregulation in many of these genes is moderate (Table S5).

In biofilms, the functional enrichment analysis of upregulated genes under permissive criteria showed no functionally enriched COG terms (Figure 3, Table S5). However, when we applied a more stringent criteria (Table 1), we found that 120 (77%) genes upregulated in biofilms are of unknown function (COG terms S) (Figure 3, Table S2). Similar to blebs, the high number of genes with unknown function among biofilm-upregulated genes showed that genetic mechanisms governing the biofilm formation are deeply understudied.

Genes that are downregulated in biofilms under permissive criteria showed enrichment of several COG functional categories (Figure 3, Table S5). For example, 66% of genes (35 out of 53 in the whole genome) labeled with the COG term M (cell wall/membrane/envelope biogenesis) are downregulated in biofilms (Figure 3, Table S2). Comparable to blebs, 47% of genes (25 out of 53 genes in the whole genome) labeled with the COG term N (cell motility) are downregulated in biofilms (Figure 3, Table S2). Like in the case of downregulated genes in blebs, when we applied a more stringent criteria, we were not able to find any enriched COG term in biofilm downregulated genes (Figure 3, Table S5).

Finally, with an aim to further characterize enriched functions among differentially expressed genes, we performed the analysis of Gene Ontogeny (GO) terms, which have finer functional resolution compared to COG terms. However, we were not able to extract any new information from GO enrichment analysis other than those recovered with COG terms (Table S7).

### 2.3. Genes Upregulated in Blebs and Biofilms Are Enriched with Plasmid-Encoded Genes

**Figure 4:**
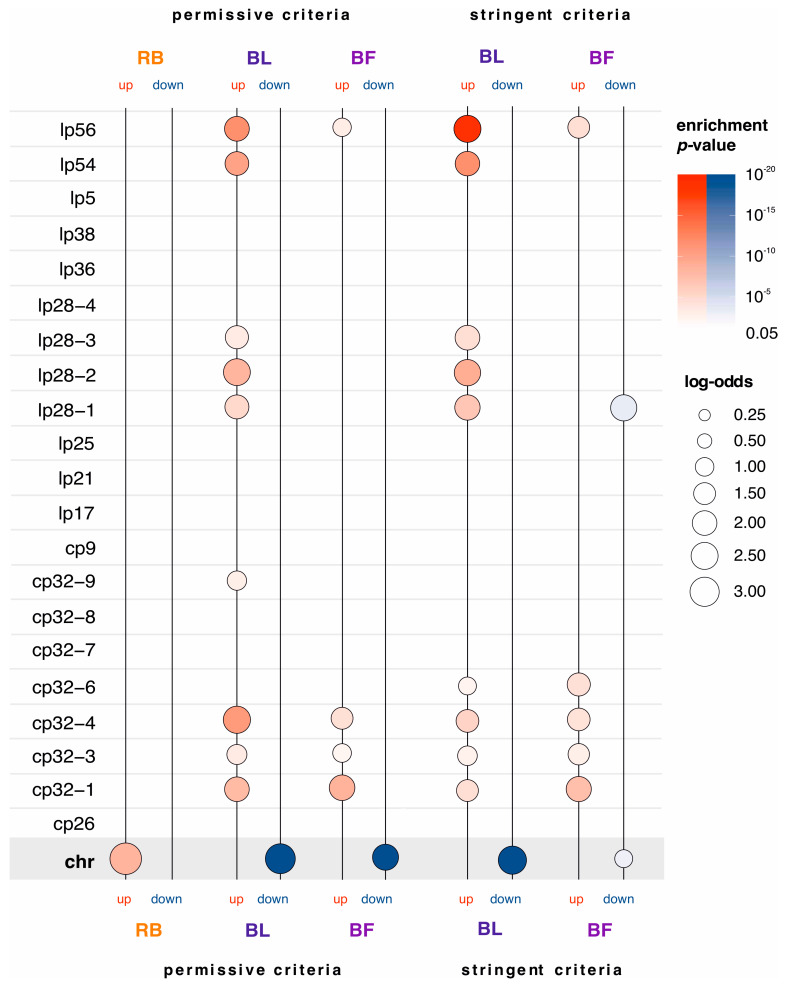
The genome localization enrichment analysis of differentially expressed genes in *B. burgdorferi* morphotypes. We showed the enrichment profiles of genome locations for upregulated (up, red color) and downregulated (down, blue color) genes that we detected in the round body (RB), bleb (BL), and biofilm (BF) dominated cultures. The leftmost column shows abbreviations for the *B. burgdorferi* B31 main chromosome (chr), circular (cp26, cp32-1, cp32-3, cp32-4, cp32-6, cp32-7, cp32-8, cp32-9, cp9) and linear plasmids (lp17, lp21, lp25, lp28-1, lp28-2, lp28-3, lp28-4, lp36, lp38, lp5, lp54, lp56). We tested the significance of enrichment by two-tailed hypergeometric test corrected for multiple comparisons at 0.05 level (Table S8). Differentially expressed genes were determined in reference to spirochetes using DeSeq2 pairwise comparisons. Under permissive criteria, we considered a gene to be differentially expressed if the shift in its expression was statistically significant (*p* < 0.05). Under stringent criteria, we additionally required that the magnitude of change was at least twofold. Under the stringent criteria, there were no differentially expressed genes in round bodies, thus the enrichment analysis was not performed. The magnitude of genome localization enrichments is depicted by log-odds (circles of different sizes) and their significance is shown in color shades (*p*-values). The gray shaded area marks significant enrichments of differentially expressed genes that reside on the main chromosome.

The genome of *B. burgdorferi* harbors, in addition a linear chromosome of about 900 kb in length, 9 circular and 12 linear plasmids [58,59]. Most genes on the main chromosome are homologs to genes with known housekeeping functions in other bacterial species [60]. On the other hand, although some plasmids carry essential genes, many genes on plasmids are coding for differentially expressed surface proteins important for the interactions between bacteria and their hosts [37,61]. To gain an insight as to where differentially expressed genes in our three morphotypes reside in the genome, we performed the enrichment analysis (Figure 4, Table S8). We found that out of 44 genes upregulated in round bodies, 43 (98%) are located on the main chromosome (Figure 4, Table S8), which emphasizes the importance of the main chromosome in the regulation of round body formation. On the other hand, genes downregulated in round bodies did not show any specific genome localization (Figure 4, Table S8).

Opposite to round bodies, in blebs and biofilms we found a pattern where most of the upregulated genes reside in plasmids (Figure 4, Table S8). Based on the permissive criteria, 369 (70%) genes upregulated in blebs and 255 (55%) genes upregulated in biofilms are located on plasmids. When we applied the stringent criteria, the number of genes located on plasmids remained high, both among genes upregulated in blebs (248, 91%) and among genes upregulated in biofilms (130, 83%). These upregulated genes are distributed on 18 out of the 21 *B. burgdorferi* plasmids (Table S8). Enrichment profiles reveal that genes upregulated in blebs are enriched on five linear plasmids (lp56, lp54, lp28-1, lp28-2, lp28-3) and five circular plasmids (cp32-1, cp32-3, cp32-4, cp32-6, cp32-9) (Figure 4, Table S8). The enrichment profile of upregulated genes in biofilms is similar to the one found in blebs, although the list of plasmids that show enrichments is shorter (lp56, cp32-1, cp32-3, cp32-6, and cp32-4) (Figure 4, Table S8).

In contrast to upregulated genes, downregulated genes in blebs and biofilms are enriched on the main chromosome (Figure 4, Table S8). Under permissive criteria, 486 (93%) genes downregulated in blebs were located on the main chromosome. Similarly, when stringent criteria are applied, 134 (94%) genes downregulated in blebs were located on the main chromosome (Figure 4, Table S8). On the other hand, 406 (88%, permissive criteria) and 45 (75%, stringent criteria) genes downregulated in biofilms were located on the main chromosome (Figure 4, Table S8). These high percentages show that the transition from spirochete to bleb and biofilm morphotypes includes an extensive shutdown of expression programs on the main chromosome. Additionally, under the stringent criteria, we found an enrichment of downregulated genes in biofilms that come from the lp28-1 plasmid (Figure 4, Table S8).

Taken together, the genome distribution of *B. burgdorferi* differentially expressed genes showed that the transition from spirochetes into round bodies is primarily associated with the upregulation of a small number of genes on the main chromosome. In contrast, transition from spirochetes to blebs and biofilms heavily relied on the shift in the expression from the main chromosome to plasmids. Interestingly, it was previously reported that outer membrane vesicles (OMVs) that shed off the bacterial surface in blebs [25,37,62] are enriched with plasmid transcripts, in contrast to the cell body where the transcripts from the main chromosome dominate [38]. However, the functional significance of this enrichment with plasmid transcripts in OMVs is unclear.

### 2.4. Biofilms and Blebs Express Evolutionary Younger Genes

**Figure 5:**
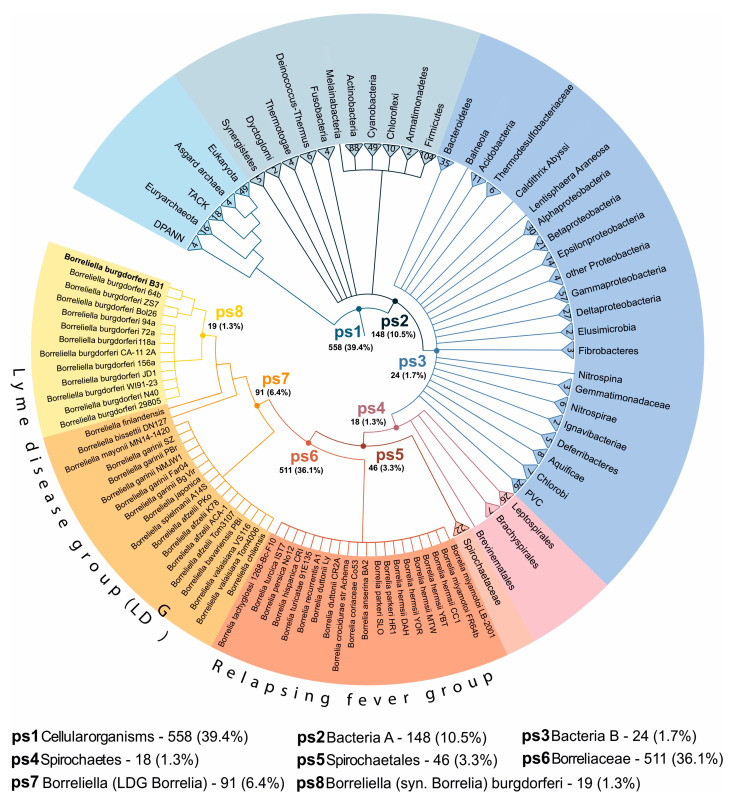
The consensus phylogeny used in the phylostratigraphic analysis. The consensus tree covers divergence from the last common ancestor of cellular organisms to *B. burgdorferi* as a focal species (see File S9 for a fully resolved tree). The tree is constructed by considering the importance of evolutionary transitions, availability of reference genomes, and their completeness estimated using BUSCO scores. The eight internodes (phylostrata) that lead from the root of the tree to the focal species (*B. burgdorferi* B31) are marked by ps1–ps8. Numbers at the top of terminal nodes represent the number of species in the fully resolved tree and correspond to the genomes used to populate the reference database for sequence similarity searches. The number of *B. burgdorferi* genes traced to each phylostratum, and a corresponding percentage, is written following the phylostratum name.

To reveal the evolutionary origin of differentially expressed genes in *B. burgdorferi* morphotypes, we performed a phylostratigraphic analysis [42,49,50,51,52]. After defining the consensus phylogeny, which contained eight internodes (phylostrata, ps) in the span from the ancestor of cellular organisms to the origin of *B. burgdorferi* (Figure 5, File S9), we successfully traced the phylogenetic origin of 1415 (99%) *B. burgdorferi* protein-coding genes using blastp sequence similarity search algorithm at the e-value threshold of 10^−3^ (Figure 5, Table S10). In our phylogeny, all known Lyme disease related *Borrelia* species cluster together in the Lyme disease group (LDG), which was recently taxonomically renamed as a new genus *Borreliella* (ps7, Figure 5, File S9). According to the new taxonomy, its sister clade, which contains all known *Borrelia* species linked to relapsing fever, remained the genus *Borrelia* [63,64,65]. However, as only recently the debate on this taxonomic split within *Borreliaceae* [63,64,65,66,67] has been resolved by International Committee on Systematics of Prokaryotes [68], we marked the respective clades with new and old taxonomic names to avoid any confusion (Figure 5, File S9). Of note, this taxonomic debate does not influence in any way our phylostratigraphic analyses because the species phylogeny we used is unaffected by naming conventions.

The obtained distribution of *B. burgdorferi* genes on the phylogeny is comparable to previous analyses of *Bacillus subtilis* [42], in that the genes in both species could be tracked to a broad range of evolutionary periods. For instance, we traced 558 (39%) *B. burgdorferi* genes to the oldest phylostratum (Cellular organisms-ps1), while the second most populated phylostratum *Borreliaceae* (ps6) harbored 511 (36%) genes (Figure 5, Table S10). In the two evolutionary youngest phylostrata *Borreliella* (LDG *Borrelia*, ps7) and *B. burgdorferi* (ps8) we found 91 (6.4%) and 19 (1.3%) genes, respectively (Figure 5, Table S10).

**Figure 6:**
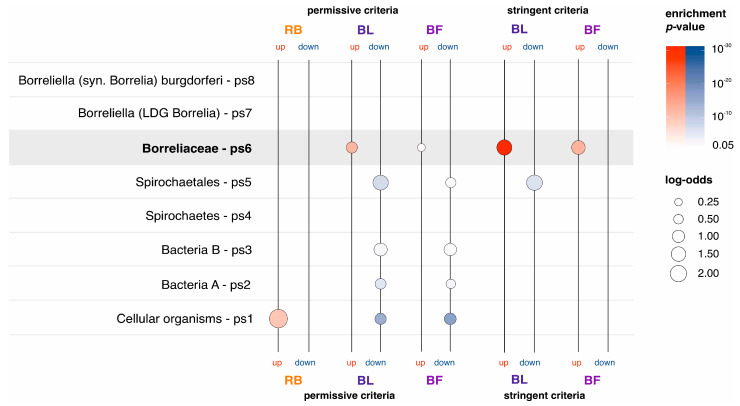
The phylostratigraphic enrichment analysis of differentially expressed genes in *B. burgdorferi* morphotypes. We showed the enrichment profiles in phylostrata along *B. burgdorferi* evolutionary lineage for upregulated (up, red color) and downregulated (down, blue color) genes that we detected in the round body (RB), bleb (BL), and biofilm (BF) dominated cultures. We tested the significance of enrichment by two-tailed hypergeometric test corrected for multiple comparisons at 0.05 level (Table S11). Differentially expressed genes were determined in reference to spirochetes using DeSeq2 pairwise comparisons. Under permissive criteria, we considered a gene to be differentially expressed if the shift in its expression was statistically significant (*p* < 0.05). Under stringent criteria, we additionally required that the magnitude of change was at least twofold. Under the stringent criteria, there were no differentially expressed genes in round bodies, thus the enrichment analysis was not performed. The magnitude of enrichments within a phylostratum is depicted by log-odds (circles of different sizes) and their significance is shown in color shades (*p*-values). Gray shaded area marks phylostratum 6 (*Borreliaceae*) where we found strong enrichment signals for bleb and biofilm upregulated genes.

To explore if differentially expressed genes in round body, bleb, and biofilm-dominated cultures show some evolutionary biases, we performed an enrichment analysis (Figure 6, Table S11). In the permissive set of round body differentially expressed genes (Table 1) we found the strong enrichment signal at Cellular organisms (ps1), which represents the evolutionary oldest phylostratum (Figure 6, Table S11). The distribution of genes on the phylostratigraphic map showed that 39 (89%) differentially expressed genes in round bodies contribute to this signal (Table S11). On the other hand, genes downregulated in round bodies did not show any evolutionary enrichment signals (Figure 6, Table S11). Like in previous analyses here, the lack of differentially expressed genes in round bodies under the stringent criteria (Table 1), precluded further enrichment analyses. These results suggest that the spirochete to round body transition heavily relies on the moderate transcriptional upregulation of evolutionary ancient genes that are common to all cellular organisms.

In bleb and biofilm morphotypes we found completely opposite evolutionary imprints compared to round bodies. In the permissive and stringent sets of upregulated genes in blebs (Table 1), we found strong enrichment signals at the origin of *Borreliaceae* (ps6) (Figure 6, Table S11). These enrichments signals are underpinned by 244 (49%) and 176 (69%) bleb upregulated genes in the permissive and stringent analyses respectively (Figure 6, Table S11). When we considered bleb downregulated genes (Table 1) we found that they are enriched with genes that are specific for Spirochaetales (ps5) (Figure 6, Table S11) in the permissive and stringent analyses. This pattern suggests that, during the morphotype transition from spirochetes to blebs, *B. burgdorferi* turns off transcription programs specific for Spirochaetales (ps5) and switches on an evolutionary younger gene set specific for *Borreliaceae* (ps6). Similar to blebs, we found that genes upregulated in biofilms are enriched with genes that originated in *Borreliaceae* (ps6) (Figure 6, Table S11). These enrichment signals are underpinned by 189 (42%) and 97 (66%) *Borreliaceae* specific genes in the permissive and stringent datasets, respectively (Figure 6, Table S11). The enrichment profile of biofilm downregulated genes is identical to the bleb profiles for the permissive dataset, but is not retained in the stringent analysis (Figure 6, Table S11). Nevertheless, these profiles suggest that spirochete to biofilm transition relies on the upregulation of genes specific for *Borreliaceae* (ps6).

To test the robustness of the obtained enrichment signals we repeated phylostratigraphic analysis in a range of blastp e-value thresholds between 1 and 10^−30^ [42] and again calculated evolutionary enrichment profiles (Table S14, Figures S1 and S2). This robustness test confirmed that our enrichment signals are fairly stable in a broad range of e-value cut-offs (Table S14, Figures S1 and S2). Taken together, our evolutionary analysis showed that the genes differentially expressed in *B. burgdorferi* morphotypes have distinct phylogenetic origin. It is striking that blebs and biofilms heavily rely on the genes that are specific for *Borreliaceae* (ps6, Table S14, Figure 6, Figure S1 and S2). Almost all species of this family, which is made of two lineages, are tick-borne pathogens of various vertebrates [65]. The family *Borreliaceae* (ps6) is a very diverged clade within the order Spirochaetales [69], which evolutionary origin is probably linked to the switch from the symbiosis with arachnid species to the biphasic parasitic lifestyle that includes arachnid and vertebrate hosts [65]. This suggests that bleb and biofilm upregulated genes, many of which emerged at the base of *Borreliaceae* (ps6, Figure 6, Table S11), might have functions that allowed adaptations to the biphasic parasitic lifestyle.

### 2.5. Many B. burgdorferi Virulence Genes Are Differentially Expressed in Blebs and Biofilms

**Figure 7:**
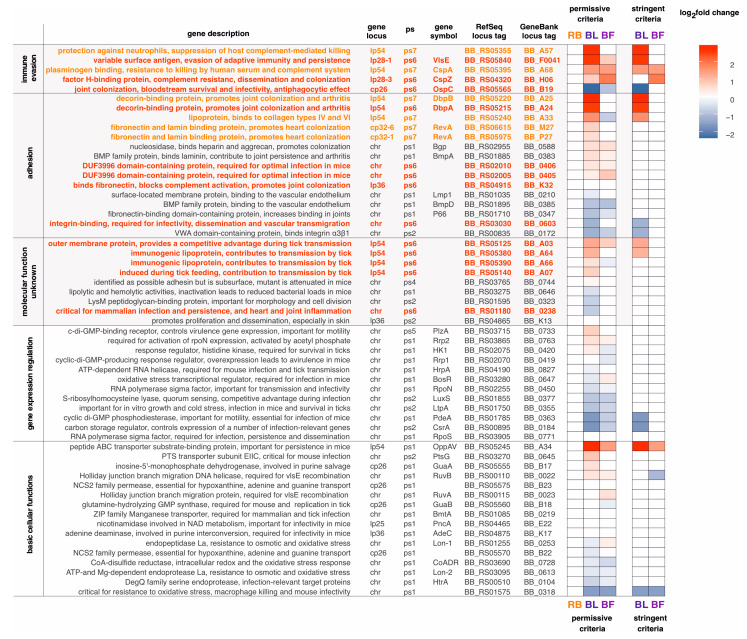
*B. burgdorferi* virulence genes are showing a morphotype-dependent transcription profile. We sorted out *B. burgdorferi* virulence genes known to be involved in Lyme disease pathogenesis [74], Table S16) and showed their normalized fold change values in round body (RB), bleb (BL), and biofilm (BF) dominated cultures in reference to spirochete-dominated cultures (Figure 2b–d, Table S4). We showed only significant fold changes. Virulence genes specific for *Borreliaceae* (ps6) are in red, while those specific for *Borreliella* (LDG *Borrelia*, ps7) are in orange. It is evident that *B. burgdorferi* immune evasion genes, which are particularly important for persistent disseminated infection, are all specific for *Borreliaceae* (ps6) or *Borreliella* (LDG *Borrelia*, ps7). Similarly, the majority of *B. burgdorferi* adhesion genes, required for dissemination and colonization of diverse tissues, are coming from these two evolutionary periods. Immune evasion and adhesion genes showed significant regulation in blebs and to lesser extent in biofilms.

Unfortunately, genes that emerged at the origin of *Borreliaceae* (ps6) are functionally extremely understudied, with 95% of them without any functional COG annotation (Table S15, Figure S3). The lack of annotation is even more severe among genes that are specific for *Borreliella* (LDG *Borrelia*, ps7) where 99% of genes have no COG function assigned. Nevertheless, it is very indicative that the function of those that are studied is linked to Lyme disease pathogenesis (Figure 7). Essentially all known *B. burgdorferi* immune evasion genes, which are particularly important for persistent disseminated infection, are specific for *Borreliaceae* (ps6) or *Borreliella* (LDG *Borrelia*, ps7) (Figure 7). For instance, we traced the evolutionary origin of the *vlsE* gene, which codes for the continuously modified surface-exposed lipoprotein (VlsE) [70], to *Borreliaceae* (ps6). In *B. burgdorferi*, VlsE undergoes antigenic variation while bacterial cells reside in the vertebrate host, and is essential for initial and persistent infection [71]. Similarly, the majority of *B. burgdorferi* adhesion genes, required for dissemination and colonization of diverse tissues, have evolutionary origin in the *Borreliaceae* (ps6) or *Borreliella* (LDG *Borrelia*, ps7, Figure 7). Examples are decorin binding proteins (DbpA and DbpB) and fibronectin-binding proteins (RevA and BBK32) which are known to be important in the dissemination and persistence of *B. burgdorferi* inside the mammalian host [72].

Taken together, this indicates that the set of functionally uncharacterized genes specific for *Borreliaceae* (ps6) and *Borreliella* (LDG *Borrelia*, ps7) likely contains undiscovered virulence genes (Table S13). Finally, the genes related to Lyme disease pathogenesis, especially those involved in immune evasion and adhesion, showed significant regulation in blebs and to a lesser extent in biofilms (Figure 7, Table S16). This suggests that bleb and biofilm morphotypes might be involved in the progression of Lyme disease [33,40,73].

## 3. Discussion

It is rather surprising that the transcriptomes of *B. burgdorferi* morphotype-dominated cultures were not previously systematically explored. This is puzzling for two reasons. First, the next generation transcriptome sequencing technology has been available for a relatively long time [75,76] and *B. burgdorferi* morphotypes were routinely grown in the laboratories [13,14,25]. Second, global transcriptome profiles are a basic-level analysis in discerning the biological relevance of different morphotypes [76]. This points to the fact that the knowledge-base on *B. burgdorferi* transcription patterns is obviously largely incomplete, which inevitably hampers the progress in Lyme disease research [14,77].

In this study, we explored the transcriptomes of three non-spirochete morphotypes that were induced by simple changes in growth conditions. However, to get a full picture of morphotype-related transcription programs in *B. burgdorferi,* the transcriptomes of morphotypes induced by alternative in vitro environmental triggers should be also investigated [12,13,14,30]. In addition, it would be very informative to perform the global protein quantification of *B. burgdorferi* morphotype-dominated cultures using the same set of environmental cues. This would yield a comprehensive overview of morphotype-related expression dynamics in *B. burgdorferi,* given that transcriptome and proteome levels are generally largely decoupled [42,55].

Another caveat relates to the fact that our morphotype cultures did not consist of entirely pure populations. The most heterogenous population was present in the bleb samples, which contained around 20% of spirochete cells without blebs. Although we tried to maximize the percentage of desired morphotypes, it is rather difficult to further reduce the remaining heterogeneity in cultures. However, the relevance of an entirely pure population is biologically questionable because it is highly unlikely that such populations exist *in vivo*. In any case, our bleb samples showed a very distinct transcriptome compared to spirochetes (Figure 1, Table 1). This suggests that if we would analyze an absolutely pure bleb population, these differences would be even more pronounced.

The bleb morphotype is currently the least studied pleiotropic form of *B. burgdorferi*. However, our analyses point to its importance for the biology of *B. burgdorferi*, because blebs showed a very distinct transcriptome which includes differential expression of many virulence genes (Figure 2 and Figure 7). However, we studied its transcriptome at only one time point; i.e., two days after we started to grow bacterial cells under aerobic conditions. At this time point exposure to aerobic conditions induces bleb formation in high percentage, which means that this morphological transformation is strongly coupled with oxygen exposure. In turn, this suggests that the bleb morphotype has some adaptive meaning for the bacterial cells under aerobic conditions. In future studies this could be improved by sampling bleb’s growth trajectory at several time points and then independently sequencing transcriptomes of these samples. This would provide much deeper understanding on the transcription dynamics that underpin this pleiomorphic form.

Similarly, we showed that *B. burgdorferi* biofilms are not a simple mix of spirochetes and blebs (Figure 1d). Their transcriptome is the most similar to bleb dominated cultures, however 30% of their differentially expressed genes do not match the bleb expression profiles. This suggest that biofilms have a unique transcriptome which should be explored in more detail in the future. In this study, we focused on morphotypes per se, and not on the developmental trajectories that lead to them. However, it would be highly interesting to sample *B. burgdorferi* biofilms along their in vitro ontogeny at several timepoints and then to recover their transcriptomes and proteomes [42]. Such a dataset would reveal temporal expression dynamics in biofilms, with the potential to uncover new coregulation patterns between *B. burgdorferi* genes. Finally, the transcription profile of round body dominated cultures was not substantially different from spirochetes. However, we explored only transcriptomes of round bodies that were sampled 30 min after this morphotype was induced by distilled water osmotic shock. Most likely, a much better picture on the transcriptional change in round bodies would be gained if samples are taken at additional time points. This suggests that further transcriptome studies of *B. burgdorferi* morphotypes are needed to reach a comprehensive understanding of their transcription profiles. In this regard, we consider our study as a starting point for future work.

Our phylostratigraphic analysis revealed that in evolutionary terms *B. burgdorferi* has a highly specialized genome. Due to its obligative parasitic lifestyle, its genome is simplified through the loss of many biosynthetic pathways [58]. This could be the result, at least in part, of functional outsourcing where an organism simplifies its genome through biological interactions [78]; in the *B. burgdorferi* case through interactions with its hosts [79]. Yet, the gene losses that lead to the strict dependence of *B. burgdorferi* on its hosts are accompanied by genome innovations linked to its parasitic lifestyle. Some of these adaptations, such as immune evasion, obviously evolved to counteract selective pressures imposed by hosts’ immune defenses.

We found that as much as 43% (621) of *B. burgdorferi* genes emerged at the origin and during diversification of *Borreliaceae* (ps6 to ps8, Figure 5). This is a noticeably higher value compared to *Bacillus subtilis* genome where we previously found that around 12% (538) of *B. subtilis* genes emerged at the origin or during diversification of *Bacillaceae* [42]. Altogether, this demonstrates that the *B. burgdorferi* genome is highly derived not only because of its simplification through extensive gene loss [58], but also due to the considerable accumulation of novel genes; i.e., orphan genes or taxonomically restricted genes [80,81,82]. These genome properties suggest that the biology and pathogenic mechanisms of *B. burgdorferi* will be evolutionary quite unique, and that the transfer of functional information via homology inference form other bacterial lineages will not be possible for many genes. It is then of no surprise that this highly diverged organism has a unique behavior and pathology that do not fit expectations largely constructed on the experience accumulated through the microbiological studies of evolutionary distant bacterial clades [14].

We showed that many *B. burgdorferi* virulence genes involved in immune evasion and adhesion evolved at the origin and during diversification of *Borreliaceae* (ps6, ps7, Figure 7). However, the vast majority of genes that evolved in these evolutionary periods have not been functionally studied, and thus their function is unknown (Figure S3). This together indicates that *Borreliaceae* specific genes, and those that emerged in younger phylostrata, (ps6–ps8, Figure 5) most likely harbor currently undiscovered Lyme disease virulence genes. A previous work on sporulation genes in *Bacillus* demonstrated that the evolutionary origin of genes is an important parameter that could be used to prioritize genes for functional analysis [83]. Given that genetic tools are available in *B. burgdorferi* [84,85], this evolutionary information opens up the possibility of narrowing down the collection of promising candidate genes for functional analyses. Taken together, we believe that many of the ongoing controversies related to Lyme disease pathogenesis and treatment strategies [14,77] could be resolved by improving our understanding of *B. burgdorferi* biology and evolution, which for unclear reasons have not yet been explored.

## 4. Methods

### 4.1. Culturing Conditions and Imaging of B. burgdorferi Pleomorphic Forms

We cultured *Borreliella* (syn. *Borrelia*) *burgdorferi* B31 (DSMZ, Brunswick, Germany, https://www.dsmz.de/collection/catalogue/details/culture/DSM-4680 accessed on 8 March 2023) in BSK-H containing 6% rabbit serum (bio&sell, Feucht, Germany) at 37 °C. We grew the typical motile *B. burgdorferi* with planar wave morphology by inoculating 40 mL BSK-H to the final concentration of 10^7^ cells/mL in 50 mL sterile and disposable conical tubes with a tightly closed lid, which created the microaerobic conditions required for growing *B. burgdorferi* [86]. After 24 h of growth in these microaerobic conditions, we collected 5 × 10^8^ cells per sample. These samples contained around 95% cells with spirochete morphotype. Our general strategy in sampling non-spirochete pleomorphic forms was to apply methods which generate the majority of cells with a specific morphotype in the shortest time. This was the optimal strategy for answering our main question: Do the transcriptomes of cultures in which round-body, bleb, or biofilm morphotype dominate differ from the transcriptome of cultures where the spirochete morphotype prevails? To obtain round body morphotype cultures, we harvested around 5 × 10^8^ spirochetes per sample by centrifugation at 5000× *g* for 5 min, resuspended them in molecular-biology grade water, and incubated them for 30 min. By applying this harsh osmotic shock, we obtained cultures where around 90% of cells had the round body morphotype. To obtain the bleb morphotype, we inoculated 6 mL BSK-H with spirochetes to the final concentration of 10^7^ cells/mL in 15 mL conical tubes with a vented lid. After 48 h of incubation under these aerobic conditions we collected around 5 × 10^8^ cells per sample. Approximately 80% of these cells had the bleb morphotype. We obtained biofilms by growing cells in 6-well tissue-culture dishes (Eppendorf, Hamburg, Germany). In each well we put 5 mL BSK-H inoculated with spirochetes to the final concentration of 10^8^ cells/mL. After 120 h of incubation, we sampled biofilm cultures for downstream analysis. We confirmed the presence of biofilms and determined their ratio against free bacteria cells by the visual observation of biofilm cultures under the microscope. We sampled only those cultures where approximately 90% of cells were located within biofilm clumps. Cells were counted using a C-Chip Disposable Haemocytometer (Neubauer Improved system, DHC-N01, Merck Millipore/Biochrom, Berlin, Germany) and a Leica DM6 B fluorescence microscope with a 40x objective using the phase-contrast (PH) setting. Different pleomorphic forms were visualized by imaging 10 µL samples under 400× magnification using a Leica DM6 B fluorescence microscope with the PH setting.

### 4.2. RNA Extraction and Sequencing

All samples were taken in three biological replicates per morphotype. All replicates contained approximately 5 × 10^8^ *B. burgdorferi* cells which we harvested by centrifugation at 5000× *g* for 5 min. The cell pellets were resuspended in 300 µL of peqGOLD TriFastTM reagent (VWR Peqlab, Darmstadt, Germany) and frozen at −20 °C. Direct-zolTM RNA Miniprep Plus Kit (Zymo Research, Freiburg, Germany) was used to extract and process RNA samples. An on-column DNA digestion was performed with the RNase-free DNase set (Qiagen, Hilden, Germany). The RNA was eluted in 50 µL of RNAse-free water and stored at −80 °C. The RNA quantity was measured spectroscopically, and the integrity was assessed by agarose gel electrophoresis.

Ribosomal RNA was removed from the total RNA samples by the Ribo-Zero rRNA Removal Kit (Illumina, San Diego, CA, USA). RNA-seq libraries were prepared using the Illumina TruSeq RNA Sample Preparation v2 Kit (Illumina, San Diego, CA, USA). Bidirectional RNA sequencing was performed on the Illumina NextSeq 500 platform at the EMBL Genomics Core Facility (Heidelberg, Germany), generating approximately 450 million reads per run. Using BBMap (V37.66) 927,047,716 paired-end sequences (75 bp) were mapped onto the *B. burgdorferi* reference genome (NCBI Assembly accession: ASM868v2; GCF_000008685.2) with an average of 94.32% mapped reads per sample (Table S1). On average, 84 million reads per replicate were mapped with low variation between the samples (Table S1). The mapping was performed using the standard settings and the option of trimming the read names after the first whitespace was enabled. The SAMtools package V2.0.3 [87] was used to generate, sort, and index BAM files for downstream data analysis. RNAseq data processing was analyzed in R V3.6.0 using custom-made scripts. Mapped reads were quantified per each *B. burgdorferi* open reading frame using the R rsamtools package V2.0.3. Raw counts for 1544 open reading frames were retrieved using the GenomicAlignments R package V1.20.1 [88]. Expression similarity across morphotypes and replicates was assessed using principal component analysis (PCA) (Figure 2a) implemented in the R package DESeq2 V1.24.0 [89] and visualized using the R package ggplot2 V3.3.2 [90] (Figure 2a).

### 4.3. Transcriptome Data Analyses and Functional Annotation

Pairwise differential gene expression between *B. burgdorferi* round body, bleb, and biofilm morphotype compared to spirochete morphotype was estimated from raw counts (1544 genes) using DESeq2 V1.24.0 package (Table S4). We performed the significance testing of differential expression by DESeq2 pipeline using Wald test [89]. The obtained *p*-values were adjusted for multiple comparisons across genes in DESeq2 pipeline using the Benjamini and Hochberg procedure [89,91]. Differences in expression between round body, bleb, and biofilm morphotypes compared to spirochetes were visualized by plotting the negative log_10_ *p*-values against log_2_ fold change values (Figure 2b–d) using the ggplot2 V3.3.2 package [90]. Two criteria were used to define which genes were considered differentially expressed. Under permissive criteria, the *p*-value had to be below 0.05 for a gene to be assigned as differentially expressed. Under the stringent criteria, in addition to *p*-value below 0.05, the fold change had to be greater than two for a gene to be assigned as differentially expressed (Tables S2 and S3). To assign functional annotation to 1544 *B. burgdorferi* genes, we searched eggNOG V5.0 database using V2 eggNOG-mapper [92] (Table S13). Clusters of Orthologous Genes (COG) and Gene Ontology (GO) functional annotations were transferred from orthologs in the Bacteria taxa (taxID:2) if the e-value was below 0.001, the bit-score was above 60, and at least 20% of the query was covered. This procedure returned a total of 635 (41.1%) genes with COG and 289 (18.7%) genes with GO annotations which are different from “unknown function”.

### 4.4. Phylostratigraphic Analyses

We performed phylostratigraphic analysis as previously described [42,49]. Following the relevant phylogenetic literature [66,69,93,94,95,96,97,98,99,100,101], we constructed a consensus phylogeny that covers the lineage from the last common ancestor of cellular organisms to the *B. burgdorferi* as a focal organism (Figure 5, File S9). We chose the nodes based on their support in phylogenetic literature, their importance in evolutionary transitions, and availability of reference genomes. We retrieved the full set of protein sequences for 743 terminal taxa, which made the reference protein sequence database, from ENSEMBL (719) and NCBI (24) databases (Table S10) and checked their completeness using BUSCO [102]. To construct the phylostratigraphic map [42,49] of *B. burgdorferi*, we compared 1425 *B. burgdorferi* protein coding genes with the reference database using the BLASTp algorithm V2.8.1 [103] and the e-value threshold of 10^−3^. We mapped 1415 protein sequences that passed phylostratigraphic procedure on the eight phylostrata of the consensus phylogeny (Table S10, Figure 6) using the previously described pipeline [42]. To test the robustness of the obtained phylostratigraphy-dependent enrichment patterns, we remapped *B. burgdorferi* protein sequences using an e-value cutoff range from 1 to 10^−30^ (Table S14, Figures S1 and S2) [42]. 

### 4.5. Enrichment Analyses

We performed all enrichment analyses using two-way hypergeometric tests [42]. In all enrichment analyses, *p*-values were adjusted for multiple comparisons using the Benjamini and Hochberg procedure [91]. We visualized enrichment analyses using custom-made scripts based on the R package ggplot2 V3.3.2 [90].

## Figures and Tables

**Table 1:** The number of differentially expressed genes in *B. burgdorferi* round body (RB), bleb (BL), and biofilm (BF) dominated cultures compared to spirochetes.

	Permissive Criteria			Stringent Criteria		
DE Cutoff	*p* < 0.05			*p* < 0.05 and Fold-Change > 2		
N (%)	Up	Down	Total	Up	Down	Total
RB	44 (2.85)	23 (1.49)	67 (4.34)	0 (0)	0 (0)	0 (0)
BL	529 (34.26)	522 (33.81)	1051 (68.07)	274 (17.75)	142 (9.20)	416 (26.94)
BF	467 (30.25)	464 (30.01)	931 (60.30)	156 (10.10)	60 (3.89)	216 (13.99)

## Data Availability

All transcriptome data have been deposited in NCBI’s Gene Expression Omnibus and are accessible through GEO Series accession number GSE199941.

## Supplementary Materials

The following supporting information can be downloaded at: https://www.mdpi.com/article/10.3390/ijms24065594/s1, Figure S1: The phylostratigraphic enrichment analysis (permissive criteria); Figure S2: The phylostratigraphic enrichment analysis (stringent criteria); Figure S3: The distribution of COG annotations; Table S1: Transcription data; Table S2: Differentially expressed genes (permissive and stringent criteria); Table S3: Differentially expressed genes in the same direction (blebs and biofilms); Table S4: Test of differential expression (all genes); Table S5: Functional enrichment analysis (COG); Table S6: Ribosomal proteins (round body); Table S7: Functional enrichment analysis (GO); Table S8: Gene localization enrichment; File S9: Full phylogeny; Table S10: Phylostratigraphic map; Table S11: Phylostratigraphic enrichment analysis; Table S13: *B. burgdorferi* gene information; Table S14: Phylostratigraphic enrichment analysis (robustness); Table S15: Annotation enrichment across phylostrata; Table S16: Known *B. burgdorferi* infection genes.
