# Supplementary material for: Pleomorphic Variants of Borreliella (syn. Borrelia) burgdorferi Express Evolutionary Distinct Transcriptomes
Source: Int J Mol Sci. 2023 Mar 15;24(6):5594. doi: 10.3390/ijms24065594 (PMC10057712; doi:10.3390/ijms24065594)
Supplement: Supplementary file 1 [file Supplementary_file_S9_v02.pdf › Supplementary_file_S9_v02.pdf]

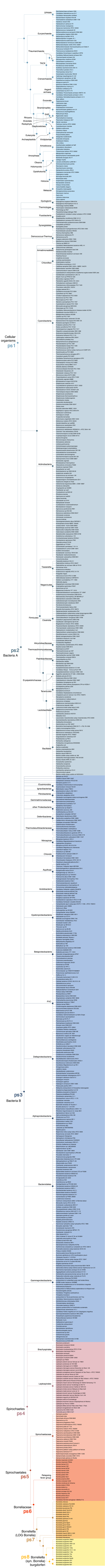
Cellular organisms
ps1
ps2
Bacteria A
ps3
Bacteria B
Spirochaetes
ps4
Spirochaetales
ps5
Borreliaceae
ps6
Borrelia
(LDG Borrelia)
ps7
ps8
Borreliella
(syn. Borrelia)
burgdorferi
