# Supplementary material for: Pleomorphic Variants of Borreliella (syn. Borrelia) burgdorferi Express Evolutionary Distinct Transcriptomes
Source: Int J Mol Sci. 2023 Mar 15;24(6):5594. doi: 10.3390/ijms24065594 (PMC10057712; doi:10.3390/ijms24065594)
Supplement: Supplementary file 1 [file ijms-24-05594-s001.zip › Supplementary_figures_S1_S3.pdf]

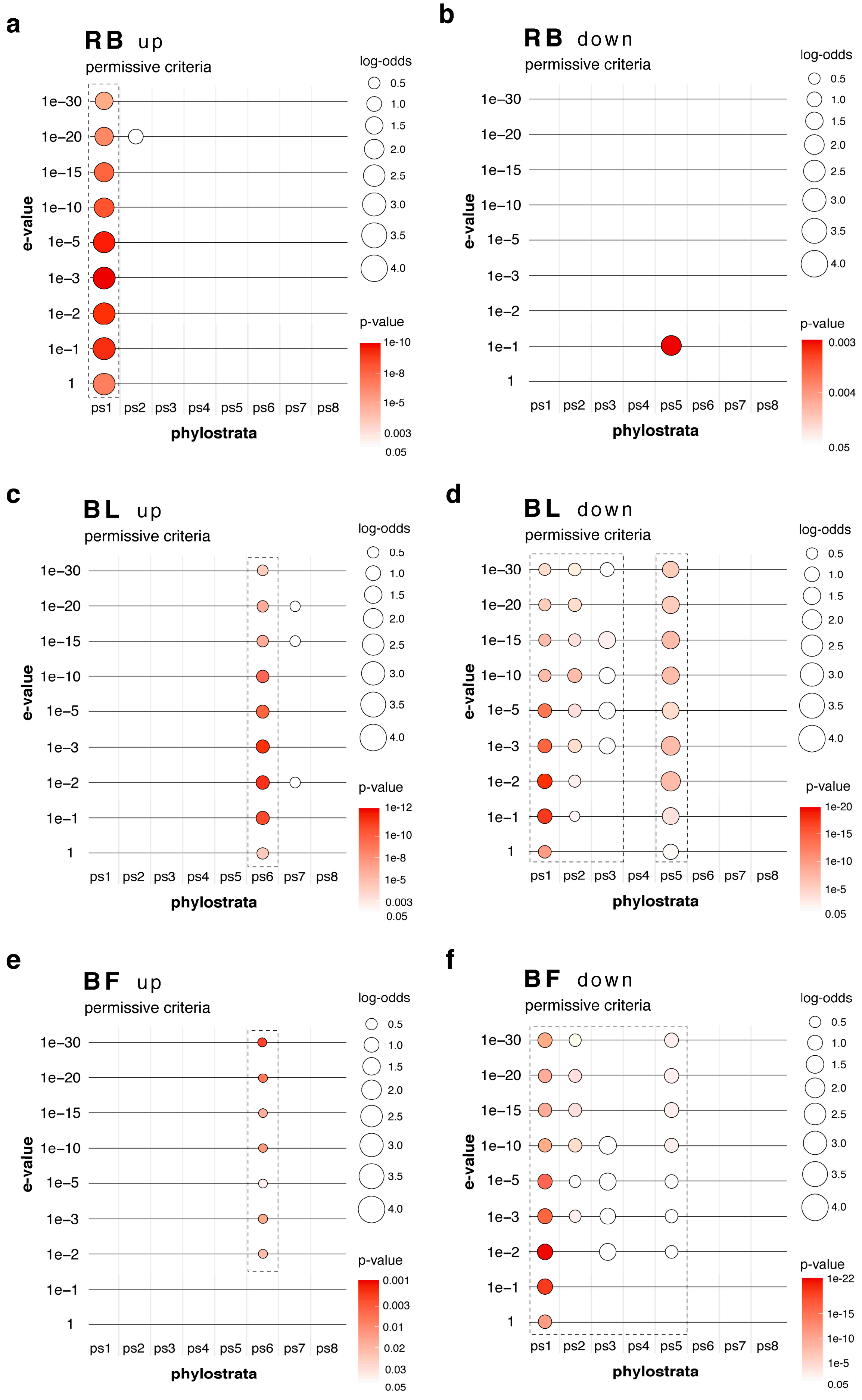
a
RB up
permissive criteria
b
RB down
permissive criteria
c
BL up
permissive criteria
d
BL down
permissive criteria
e
BF up
permissive criteria
f
BF down
permissive criteria
e-value
1e−30
1e−20
1e−15
1e−10
1e−5
1e−3
1e−2
1e−1
1
ps1 ps2 ps3 ps4 ps5 ps6 ps7 ps8
phylostrata
log-odds
0.5
1.0
1.5
2.0
2.5
3.0
3.5
4.0
p-value
1e-10
1e-8
1e-5
0.003
0.05
0.003
0.004
0.05
1e-12
1e-10
1e-8
1e-5
0.003
0.05
1e-20
1e-15
1e-10
1e-5
0.05
0.001
0.003
0.01
0.02
0.03
0.05
1e-22
1e-15
1e-10
1e-5
0.05

Figure S1. The phylostratigraphic enrichment analysis of differentially expressed genes in *B. burgdorferi* morphotypes based on permissive criteria using different e-value thresholds. We showed the enrichment profiles in phylostrata along *B. burgdorferi* evolutionary lineage for upregulated (up) and downregulated (down) genes that we detected in the round body (RB), bleb (BL), and biofilm (BF) morphotypes (Supplementary Table S14). The vertical grid represents used e-value thresholds (1, 10^{-1}, 10^{-2}, 10^{-3}, 10^{-5}, 10^{-10}, 10^{-15}, 10^{-20}, 10^{-30}). We tested the significance of enrichment by two-tailed hypergeometric test corrected for multiple comparisons at 0.05 level. Differentially expressed genes were determined in reference to spirochetes using DeSeq2 pairwise comparisons using the permissive criteria. Under permissive criteria, we considered a gene to be differentially expressed if the shift in its expression was statistically significant ($p < 0.05$). The magnitude of phylostratum enrichments is depicted by log-odds (circles of different sizes) and their significance is shown in color shades (p-values).

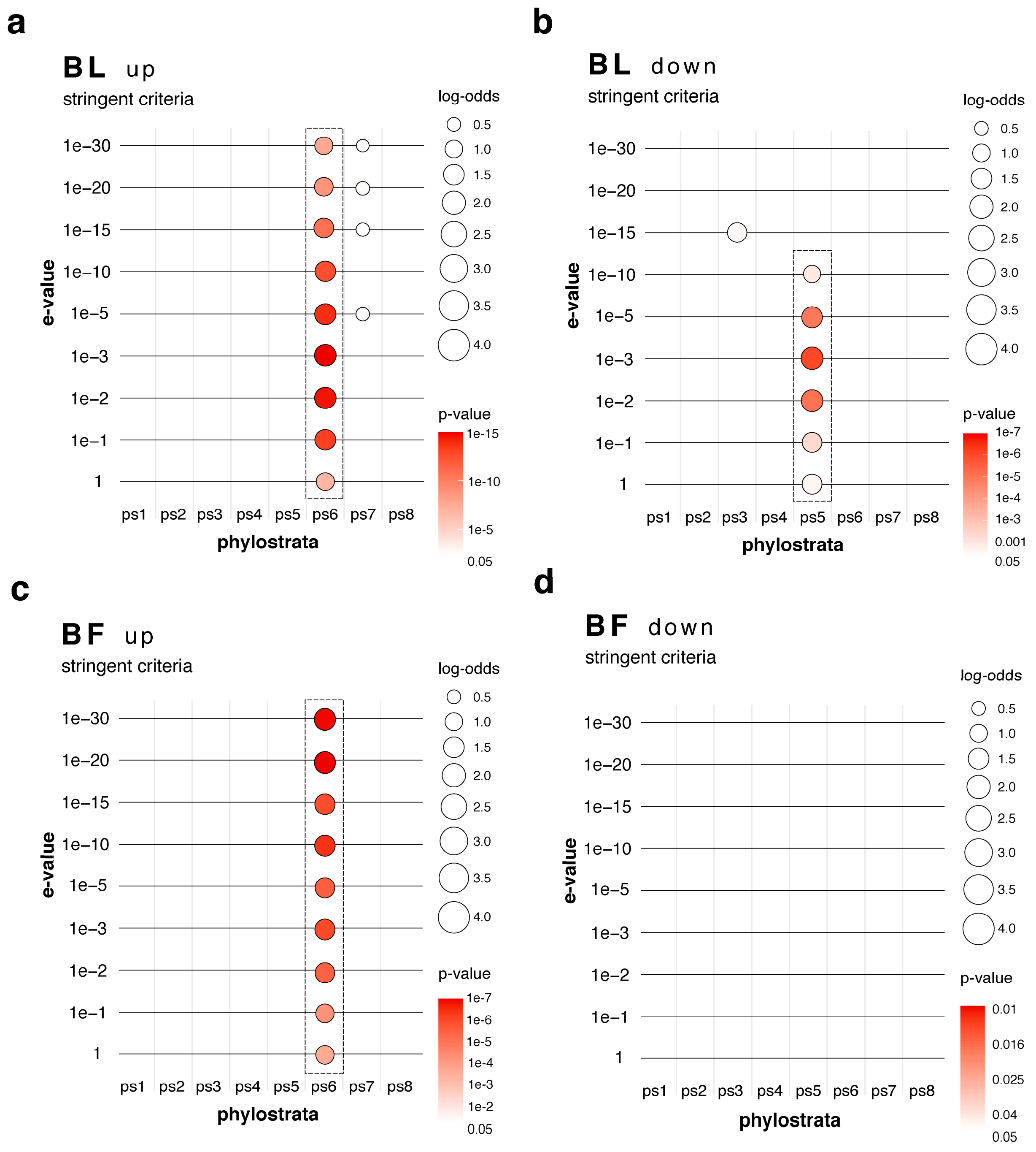

Figure S2. The phylostratigraphic enrichment analysis of differentially expressed genes in *B. burgdorferi* morphotypes based on stringent criteria using different e-value thresholds. We showed the enrichment profiles in phylostrata along *B. burgdorferi* evolutionary lineage for upregulated (up) and downregulated (down) genes that we detected in the round body (RB), bleb (BL), and biofilm (BF) morphotypes (Supplementary Table S14). The vertical grid represents used e-value thresholds (1, 10^{-1}, 10^{-2}, 10^{-3}, 10^{-5}, 10^{-10}, 10^{-15}, 10^{-20}, 10^{-30}). We tested the significance of enrichment by two-tailed hypergeometric test corrected for multiple comparisons at 0.05 level. Differentially expressed genes were determined in reference

to spirochetes using DeSeq2 pairwise comparisons and the stringent criteria. Under stringent criteria, a gene was considered differentially expressed if the shift in expression, regardless of its magnitude, was statistically significant ($p < 0.05$) and the magnitude of change was at least twofold. Under the stringent criteria, there were no differentially expressed genes in round bodies, thus the enrichment analysis was not performed. The magnitude of phylostratum enrichments is depicted by log-odds (circles of different sizes) and their significance is shown in color shades (p-values).

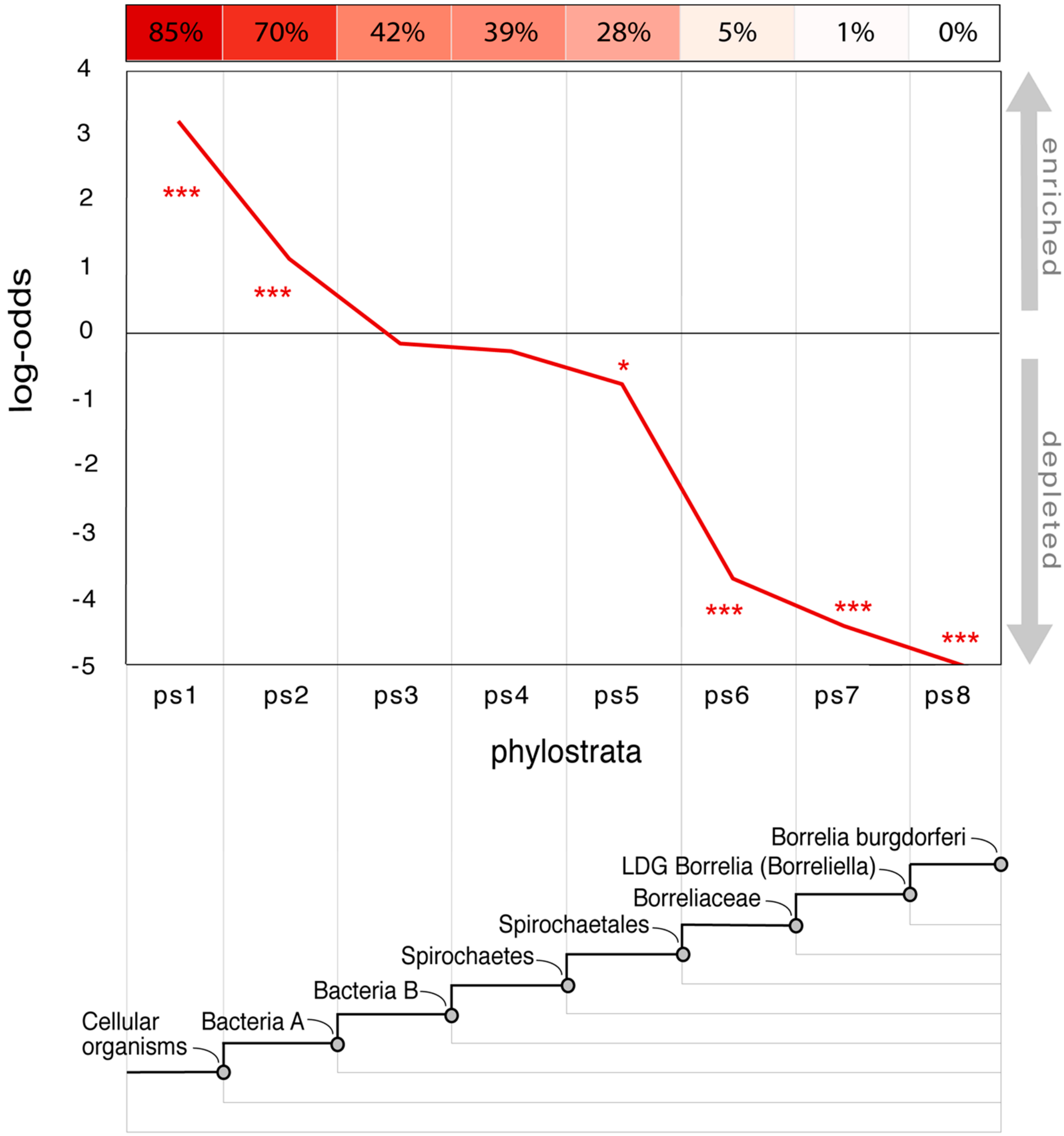

Figure S3. The distribution of COG annotated *B. burgdorferi* B31 genes across phylostrata shows a lack of the annotation of younger genes. We showed the enrichment profiles in phylostrata along *B. burgdorferi* evolutionary lineage for all COG annotated genes (Supplementary Table S13). We tested the significance of enrichment by two-tailed hypergeometric test corrected for multiple comparisons at 0.05 level (supplementary table S15). The magnitude of phylostratum enrichments is depicted by log-odds (y-axis) and their significance is shown by the number of asterisk signs (*p < 0.05; **p < 0.01; ***p < 0.001). The heatmap above the coordinate grid shows the percentage of COG annotated *B. burgdorferi* B31 genes in each phylostrata.
